# Endometrial cancer—how many patients could benefit from sentinel lymph node dissection?

**DOI:** 10.1186/s12957-018-1392-8

**Published:** 2018-05-17

**Authors:** Sarah Brugger, Moritz Hamann, Marc Mosner, Michaela Beer, Michael Braun, Martin Pölcher

**Affiliations:** 1 Rotkreuzklinikum München, Frauenklinik Taxisstraße 3, 80637 München, Germany; 2 Pathologie Rotkreuzklinikum, Winthirstraße 11, 80639 München, Germany

**Keywords:** Risk groups, Ultrastaging, Detection rate

## Abstract

**Background:**

Sentinel lymph node dissection (SLND) may reduce morbidity in patients with endometrial cancer. The objective of this study is to estimate how many systematic lymph node dissections (LND) can be spared with an implementation of a SLN-procedure.

**Methods:**

Retrospective, single-center study, SLND according to NCCN-Guidelines.

**Results:**

In 109 patients of 154 consecutive patients, SLND was performed. The detection rate was 61% on both sides and 86% on at least one side. Classification of uterine risk factors is as follows: low risk 53, intermediate risk 25, high-intermediate risk 13, and high-risk 18. Stage IIIC: 0, 3, 7, 11, respectively. Under the assumption that 56 patients with “higher than low risk” factors would be treated by systematic LND, we spared 26 pelvic and paraaortic LND. After failures of SLN detection, unilateral pelvic LND was performed in 15 patients. Patients with “higher than low risk” factors and node-negative SLN are candidates for a randomized study to prove safety and efficacy. Only every third patient in our study met these criteria.

**Conclusions:**

In a cohort of patients with “higher than low risk” endometrial cancer, the implementation of SLND nearly divided the number of radical lymph node dissections in half. Further studies are required to define the best modalities for SLND.

## Background

Endometrial cancer is the most common carcinoma of the female genital tract with over 300,000 new cases diagnosed each year worldwide [1]. Comprehensive surgical staging in endometrial cancer, i.e., hysterectomy, bilateral salpingo-oophorectomy, and lymph node assessment by pelvic and paraaortic lymph node dissection (LND), has been controversial for many years. Patients with lymph node metastasis have significant lower median survival rates compared to patients with a tumor confined to the uterus [2]. The finding of nodal involvement therefore is one of the most important diagnostic factors to initiate adjuvant treatment*.*

The probability of lymph node metastases can be determined by tumor characteristics like histological subtype, grading, lymph vessel space invasion, and myometrial invasion (Table 1). A risk-adopted management of LND according to current consensus statements provides a simple hysterectomy without lymph node assessment in patients with uterine low-risk factors and a systematic lymph node dissection in patients with high-risk factors [3]. In patients with intermediate-risk factors or high-intermediate-risk factors, the management is weighting of risks, and the best practice is not yet established.

**Table 1:** Risk groups according to ESMO-ESGO-ESTRO Consensus Conference [3]

	Grading	Histological type	Stage	LVSI
Low risk	G1, G2	Endometrioid	IA	Negative
Intermediate risk	G1, G2	Endometrioid	IA	Positive
	G1, G2	Endometrioid	IB	Negative
	G3	Endometrioid	IA	Negative
High-intermediate risk	G3	Endometrioid	IA	Positive
	G1, G2	Endometrioid	IB	Positive
High risk	G3	Endometrioid	IB	Negative/positive
	G3	Non-endometrioid	IA/B	Negative/positive
			II	Negative/positive

Stages *IA* myometrial invasion < 50%, *IB* myometrial invasion ≥ 50%; stage *II* cervical involvement; *LVSI* lymphovascular space invasion

In gynecologic cancers, LND can cause severe long term-morbidity, especially lymph edema of the legs and should be omitted whenever appropriate [4, 5]. Preoperative imaging does not correspond well with postoperative findings [6]. Therefore, surgical staging remains the gold standard in the assessment of lymph node status. Systematic lymph node dissection was a dogma of cancer surgery in the past century. Its role as a therapeutic procedure has become controversial in different cancer types, e.g., breast and ovarian cancer [7–10], especially when adjuvant treatment follows lymph node surgery.

Sentinel lymph node dissection (SLND) can serve as a useful tool whenever reduction of morbidity can be achieved without lacking the information concerning nodal involvement, adjuvant treatment, and prognosis. Sentinel lymph node mapping as introduced by Abu-Rustum means removal of any colored or suspicious lymph nodes. This might be a common ground between no lymph node assessment and radical pelvic and paraaortic LND [11, 12].

To prove efficacy and safety of this approach, a prospective and randomized trial would be appropriate. Two randomized studies in endometrial cancer could not show a benefit for patients who have undergone systematic LND in recurrence-free survival (RFS) or overall survival (OS) compared with patients without LND [13, 14]. Three major concerns have been raised over these data. Firstly, a great proportion of patients with uterine low-risk factors may have led to the concealment of a survival difference in patients who were at higher risk. Secondly, the lymph node assessment like sampling and bulky node dissection in patients randomized for omitting lymph node assessment may have caused a crossover effect. And lastly, the lack of standardized adjuvant treatment regimens led to an unequal distribution of patients who had adjuvant radiotherapy or chemotherapy [15].

The aim of this study was to incorporate a sentinel lymph node (SLN) procedure with a special focus on the proportion of patients who could benefit most from this procedure: how many patients have a negative sentinel lymph node and are at a higher risk than low risk, i.e., intermediate risk, high-intermediate risk, or high risk? In this group, a systematic LND can be omitted by the implementation of a reliable SLN procedure. To prove safety and efficacy within a clinical trial, this “higher than low risk” group would serve as potential target population for a phase 3 protocol. We tried to determine the feasibility of a study with regard to the experience of the former studies.

## Methods

In this retrospective single-center study, lymph node assessment by SLND was offered in all assumed stage I cancers and registered in a prospectively maintained database. Inclusion criteria for this study were histologically confirmed endometrial cancer by dilation and curettage (D/C) and disease confined to the uterine corpus in transvaginal ultrasound imaging (stage I disease). Patients had to be deemed fit for major abdominal surgery and potential pelvic and paraaortic lymph node dissection.

Exclusion criteria were known allergic reaction to patent blue dye, node positive, or locally advanced disease in previous imaging. Computer tomography or magnetic resonance imaging was not mandatory; however, if patients presented to our clinic with preoperative imaging, they were excluded if locally advanced disease or lymph node metastasis was suspected. One surgeon performed the first 50 SLND and two further surgeons the SLND of the entire population. Informed consent was obtained from all individual participants included in the study.

A total of 4-ml patent blue dye was injected into the cervix at 3 and 9 o’clock (1-mm and 1-cm depth, 1 ml respectively) at the beginning of surgery according to the National Comprehensive Cancer Network (NCCN) guidelines [16]. Detection and removal of sentinel lymph nodes was then performed for each side. If detection of a SLN failed, no definitive proceeding was stated. This means that pelvic and paraaortic LND, pelvic LND on one or both sides, or no further lymph node assessment was performed at the discretion of the surgeon. If detection of sentinel lymph node failed, pelvic and paraaortic LND, pelvic LND on one or both sides, or no further lymph node assessment was performed at the discretion of the surgeon. In patients with short history of postmenopausal vaginal bleeding, inconspicuous ultrasound findings, good differentiation after D/C, lack of lymphovascular space invasion, or atypical hyperplasia with transition into a well differentiated carcinoma, a great probability of a low-risk situation was to assume. In these cases, pelvic LND was omitted even if the SLN was not detected. LND was performed within a second operation if the final histopathological reports showed uterine risk factors “higher than low risk”. In patient with supposed uterine risk factors “higher than low risk” (e.g., suspected MI > 50% in ultrasound diagnostic, G3/type2/LVSI in the D/C specimen), consecutive lymph node assessment was performed within the same operation.

For better detection of low volume metastasis, ultrastaging of all SLN was performed by additional serial sectioning and immunohistochemical staining. Definition of lymph node micrometastasis was 0.2–2.0 mm and ITC up to 0.2 mm.

## Results

Between February 2013 and February 2017, a total of 154 patients with endometrial cancer were treated by surgery at our institution. A total of 45 patients (29%) were ineligible for SLND: Advanced disease was suspected in 16 patients (local advanced disease (1)/lymph node metastases (8)/distant metastases (7)). General or geriatric assessment deemed 10 patients with a median age of 85 years to be unsuitable for LND. In 9 patients, previous hysterectomy showed unexpected carcinoma; therefore, the opportunity for sentinel mapping was missed. Refusing written informed consent, 10 patients were excluded, the main reason being due to having concerns about lymph node surgery.

SLND was performed in 109 patients. Patient characteristics and risk assessments are shown in Table 2. Overall, node-positive disease was detected in 19% (*n* = 21). In the low-risk group, no lymph node metastases were detected; in the intermediate 12% (*n* = 3); in the high-intermediate-risk group 54% (*n* = 7); and in the high-risk group 61% (*n* = 11). A change of the grading score after hysterectomy—in contrast to the tumor characteristics available by curettage specimens—occurred in 14% of patients (*n* = 15).

**Table 2:** Patient characteristics of 109 SLND

Age, years (range)	Median	63.5	(28–89)
Grading, uterus-specimen	G1	57	52%
	G2	30	28%
	G3	22	20%
Histopathological type			
Type I		99	91%
Type II		10	9%
	Serous		6
	Clear cell		1
	Carcinosarcoma		3
Uterine risk factors	Low risk	53	49%
	Intermediate risk	25	23%
	High-intermediate risk	13	12%
	High risk	18	17%
Nodal involvement	Low risk	0	0%
	Intermediate risk	3	12%
	High-intermediate risk	7	54%
	High risk	11	61%
Final FIGO stage	IA	60	55%
	IB	23	21%
	II	5	5%
	IIIC1	10	9%
	IIIC2	5	5%
	IIIA	1	1%
	IVB	5	5%

The detection rate of the sentinel node was 61% (*n* = 67) on both sides and 86% (*n* = 94) on at least one side. The detection on the right pelvic side succeeded in 86 patients, on the left pelvic side in 74 patients. On average, 2.4 lymph nodes were removed on the right side and 2.0 lymph nodes on the left side. In 4 patients (3.7%) with “higher than low risk” tumors, the lymph node metastases were detected by ultrastaging of the sentinel nodes. These patients may have been falsely classified as lymph node negative with conventional staining methods if a lymph node dissection would have been chosen. In addition, isolated tumor cells (ITCs) were found in 4 out of 94 patients (4.3%).

A risk-adopted management would have caused pelvic and paraaortic LND in all patients “higher than low risk” (*n* = 56). In this study, we performed 109 SLN procedures. We performed 30 pelvic and paraaortic LND and 15 pelvic unilateral LNDs (8 of them were low-risk patients). In 65 patients, no further lymph node dissection was performed. In 38 cases, LND was performed simultaneously and in 7 cases within a second operation. Management after failure of sentinel detection is shown in Table 3.

**Table 3:** Management of lymph node dissection after injection of blue dye for sentinel lymph node dissection

	Low risk		Intermediate risk		High-intermediate risk		High risk		Sum	
	*n*		*n*		*n*		*n*			
SLN detection failed on both sides	7		4		0		4		15	
No further dissection		7		1				0		8
Pelvic LND		0		0				0		0
Pelvic and para-aortic LND		0		3				4		7
SLN detection failed on one side	13		3		5		6		27	
No further dissection		6		0		0		2		8
Pelvic LND		7		2		3		0		12
Pelvic and para-aortic LND		0		1		2		4		7
SLN detection successful on both sides	33		18		8		8		67	
No further dissection		33		11		6		1		51
Pelvic LND		0		0		0		0		0
Pelvic and para-aortic LND		0		7		2		7		16
Sum	53		25		13		18			109

*SLN* sentinel lymph node, *LND* lymph node dissection

After successful detection of SLN on both sides, 16 pelvic and paraaortic LND identified 12 node-positive and only four node-negative patients. The lymph node status was correctly predicted by SLND in all 16 cases. In this small subgroup, the false-negative value was 0%.

In one patient with high-intermediate risk, we found isolated paraaortic lymph node metastases after failure of sentinel detection on one pelvic side; 3 of 63 lymph nodes were involved. Only one SLN was found in the paracaval region and was negative.

Only one patient in the “higher than low risk” group with stage IB G1 had no lymph node assessment on both sides due to having no detection of a SLN on one side and refusing a second operation for systematic LND.

Of 109 patients, 56 tumors were “higher than low risk”. In 34 patients of this cohort, no lymph node involvement was found. These patients represent the group who may benefit most from a sentinel lymph node dissection as systematic dissection can be omitted.

In two patients, a conversion from laparoscopy to open surgery occurred for intraoperative bleeding or unexpected disseminated peritoneal carcinomatosis. In one patient, there was a thermic lesion at the appendix with consecutive appendectomy. In another patient, an ileum perforation occurred with consecutive bowel resection after laparoscopic LND.

Postoperative complications within 28 days included the following: transitory weakness/numbness of the legs (*n* = 3), vaginal bleeding or hematoma of the vaginal vault (*n* = 3), trocar hernia with consecutive surgery (*n* = 2), surgical site infection after laparotomy (*n* = 5), transfusion for postoperative anemia (*n* = 2), and mechanically ileus (*n* = 1). There were no significant differences between patients with hysterectomy and SLND compared to those with pelvic or pelvic and paraaortic LND.

## Discussion

The current concept of lymph node assessment follows a *risk-adopted* algorithm. It suggests a systematic LND in patients with endometrial cancer and uterine high-risk factors. In these patients, the incidence of lymph node involvement is high, as seen in this trial with 61% node-positive patients. For patients with low-risk factors, lymph node assessment may be omitted. In this study, neither lymph node metastases nor micrometastases (MM) were found after SLND in 53 low-risk patients—even after a thorough examination, so called ultrastaging.

In patients with intermediate and high-intermediate-risk factors, the significance of LND is not clear [3]. The proportion of these two groups, however, was 35% of the entire patient cohort, and we found lymph node metastases in 12% of the intermediate-risk group and 54% in the high-intermediate-risk group. This indicates that lymph node assessment is potentially meaningful for these patient groups [17].

A weakness of the current *risk-adopted* lymph node management is that the proper risk group cannot safely be determined at the time of surgery: depth of invasion, grading, lymph vessel invasion, and cervical involvement are factors needed for the indication of a LND. Intraoperative pathology is not always a reliable method for determining these factors [18–21]. In this study, grading differed in 14% comparing curettage specimen with hysterectomy specimen. Therefore, patients may suffer a second operation if risk factors change after hysterectomy and a systematic LND is recommended.

In contrast, the *SLN procedure* is used in all patients, regardless of special risk factors. Detection can only take place before removing the uterus because blue dye tracer colors lymph vessels and nodes only temporarily. Therefore, a high number of rather optional SLND in low-risk patients has to be accepted. The concept of SLND therefore introduces a totally different approach. The most challenging issue in SLND is the surgical management in case of non-detection of a SLN. As a midline organ, uterine lymphatic vessels drain on both pelvic sides and therefore detection of SLN on both sides is recommended. The NCCN-algorithm provides for systematic pelvic LND on the side, where the SLN is not detected. This enables a one-stop strategy, and therefore, a second operation is not intended.

We did both: immediate consecutive LND in cases of non-detection of the SLN, especially in patients with known risk factors “higher than low risk” and secondary operation with LND, e.g., in patients with a positive ultrastaged SLN. To detect 21% node-positive patients, we performed lymph node assessment with at least one pelvic LND in 41% of the entire cohort of SLN patients. In 30 patients, a pelvic and paraaortic LND was performed. Assumed that pelvic and paraaortic LND would be reasonable for all 56 patients *higher than low risk*, we nearly divided the number of radical lymph node dissections in half.

Reconsidering the main critical comments on former lymph node dissection trials, a study with a high proportion of low-risk patients might not be suitable to show the equivalence of different surgical lymph node assessments [15]. Therefore, a trial should only include patients of the “higher than low-risk” group. A comparison of both strategies, “risk adopted” vs. “SLN mapping,” would be one protocol option. Another option could be a study providing for LND in cases of positive SLN and in cases of negative SLN with a randomization into systematic LND or observation. As shown in this study, a feasible randomized trial to measure safety and outcome of SLND versus LND requires an exceedingly large number of patients as only approximately every third patient undergoing SLND would be eligible for randomization.

A study comparing outcome of surgical procedures should predefine adjuvant treatment. Regimens for patients with higher risk of recurrence or node-positive disease with either chemotherapy, radiation therapy, or both are being investigated in several clinical trials (PORTEC 1, GOG 99, PORTEC-2, GOG 122, GOG177, RTOG 0763) [22–26]. Recently, first results of a phase 3 trial (PORTEC-3) addressing the benefit of adjuvant chemotherapy during and after radiotherapy versus pelvic radiotherapy alone for women with high risk and advanced endometrial cancer were reported. No difference was found in progression-free survival (PFS) [27]. However, in the subgroup of all stage III patients including those with nodal involvement, there was a significant benefit in the 5-year PFS for the combined regimen (69.3 vs. 58.0% [95% CI 0.45–0.97, *p* = 0.032]). This study can help to determine a standardized adjuvant treatment within a protocol.

To date, there are no prospective studies demonstrating a survival benefit for a systematic pelvic and paraaortic LND. It is questionable whether a clinical study could find a difference between *two types* of lymph node assessments, assuming there is one. The risk of missed paraaortic metastases within the SLN procedure could be offset by the higher detection rate of stage III disease by ultrastaging. Hence, a protocol for a large-scale validation trial of lymph node management is a challenge.

Technically, the implementation succeeded without any problems: We instantly achieved a detection rate that is exactly on par with the literature [28–30]. Several studies were reported to improve detection rates with different tracers or injection sites. A new technique utilizes indocyanin-green (ICG) and the fluorescence signal can be detected with a special infrared sensor [31, 32]. Recent studies point out that ICG might develop as a new standard in detection of sentinel lymph nodes due to the fact it shows better detection rates, especially in patients with a high body mass index [33]. Laparoscopic injection into the uterine fundus has not been established, as detection rates were low. The main criticism of the cervical injection site is a possible lack of representative mapping in the paraaortic area. In a retrospective analysis, a survival benefit for patients with paraaortic lymph node dissection in endometrial cancer was described, indicating that nodal involvement in this area is clinically meaningful [34]. In one patient, after failure of SLN detection, we found a paraaortic lymph node metastasis without pelvic positive nodes. These isolated paraaortic metastases are rare with rates ranging from 1 to 1.5% [35–37].

Ultrastaging of sentinel lymph nodes leads to a higher detection rate of low-volume lymph node metastases [38]. In our study, metastases were detected by ultrastaging in 4% of the cases which were not detected by conventional histology. This number is even lower than published in literature with upstaging as a result of ultrastaging in up to 20% [39–41], though the prognostic value of low-volume lymph node metastases and the best management of this finding are not yet established. Patients with ITC and MM received adjuvant chemotherapy more frequently and showed a better oncologic outcome when compared to patients with macrometastasis over a time period of 3 years [40, 42]. Bezu et al. found a link between the occurrence of MM and higher recurrence rate of uterine cancers [43].

A prospective multicenter cohort study with indocyanine green in 340 patients identified 35 of 36 patients with lymph node metastases by SLN mapping and consecutive dissection, yielding a sensitivity of 97.2%, a negative predictive value of 99.6% and a false-negative rate of 3% [29]. In a meta-analysis which included 4915 women, Smith et al. concludes that sentinel mapping accurately predicts nodal status in women with endometrial cancer and may be considered an alternative standard of care in the staging of women with endometrial cancer [30]. Holloway et al. raise the question, whether SLND is the more precise method to target lymph node involvement [44].

Several limitations of this study should be emphasized. We did not perform LND after SLND in generally and therefore cannot report our false-negative rate. Preoperative imaging targeting myoinvasion or lymph node status was not standardized. Therefore, the assumption of stage I disease was made on different basic principals in the screening of 154 patients. Deep myometrial or cervical invasion in magnetic resonance imaging may have influenced decisions concerning lymph node management. No fixed algorithm after non-detection of sentinel nodes was provided; in some patients, we temporized the final histopathologic report to estimate final risk factors, for example in patients with supposed low-risk features.

A withdrawal of radical lymph node surgery was recently observed in breast and ovarian cancer [7, 10]. Therefore, several questions have to be addressed in the future of endometrial cancer treatment. It is not proven that systematic LND is mandatory in each case of positive SLN, especially when adjuvant treatments are scheduled. The role of lymph node status in order to guide postoperative management may be replaced at some point by other prognostic factors, e.g. CD171 [45]. Genetic profiling showed different subtypes after next generation sequencing in endometrial cancer. This may help to identify more adequate candidates for adjuvant treatment in terms of targeted therapies [46, 47].

## Conclusion

Current surgical practices in endometrial cancer today range from no assessment of lymph nodes to comprehensive lymph node dissection. Both strategies are at high risk of over- and undertreatment. Sentinel lymph node dissection in endometrial cancer is a promising technique and, therefore, a step towards individualized treatment. Systematic pelvic and paraaortic LND can be minimized. However, safety data are lacking. A requested randomized trial poses a challenge in protocol design and patient recruitment.

## Abbreviations

D/C: Dilatation and curettage
ITC: Isolated tumor cells
LND: Lymph node dissection
MM: Micrometastases
NCCN: National Comprehensive Cancer Network
SLN: Sentinel lymph node
SLND: Sentinel lymph node dissection
